# Beta Diversity in a Highly Heterogeneous Area: Disentangling Species and Taxonomic Dissimilarity for Terrestrial Vertebrates

**DOI:** 10.1371/journal.pone.0160438

**Published:** 2016-08-08

**Authors:** Jaime M. Calderón-Patrón, Irene Goyenechea, Raúl Ortiz-Pulido, Jesús Castillo-Cerón, Norma Manriquez, Aurelio Ramírez-Bautista, Alberto E. Rojas-Martínez, Gerardo Sánchez-Rojas, Iriana Zuria, Claudia E. Moreno

**Affiliations:** Centro de Investigaciones Biológicas, Instituto de Ciencias Básicas e Ingeniería Universidad Autónoma del Estado de Hidalgo, Pachuca, Hidalgo, Mexico; University of Rouen, France, FRANCE

## Abstract

Quantifying differences in species composition among communities provides important information related to the distribution, conservation and management of biodiversity, especially when two components are recognized: dissimilarity due to turnover, and dissimilarity due to richness differences. The ecoregions in central Mexico, within the Mexican Transition Zone, have outstanding environmental heterogeneity and harbor huge biological richness, besides differences in the origin of the biota. Therefore, biodiversity studies in this area require the use of complementary measures to achieve appropriate information that may help in the design of conservation strategies. In this work we analyze the dissimilarity of terrestrial vertebrates, and the components of turnover and richness differences, among six ecoregions in the state of Hidalgo, central Mexico. We follow two approaches: one based on species level dissimilarity, and the second on taxonomic dissimilarity. We used databases from the project “Biodiversity in the state of Hidalgo”. Our results indicate that species dissimilarity is higher than taxonomic dissimilarity, and that turnover contributes more than richness differences, both for species and taxonomic total dissimilarity. Moreover, total dissimilarity, turnover dissimilarity and the dissimilarity due to richness differences were positively related in the four vertebrate groups. Reptiles had the highest values of dissimilarity, followed by mammals, amphibians and birds. For reptiles, birds, and mammals, species turnover was the most important component, while richness differences had a higher contribution for amphibians. The highest values of dissimilarity occurred between environmentally contrasting ecoregions (i.e., tropical and temperate forests), which suggests that environmental heterogeneity and differences in the origin of biotas are key factors driving beta diversity of terrestrial vertebrates among ecoregions in this complex area.

## Introduction

Biodiversity is the result of a wide variety of factors, among which historical biogeographical processes, and environmental heterogeneity play major roles [1], therefore there is a special concern in disentangling the causes of high biodiversity in heterogeneous regions with a complex history of its biota. Beta diversity is the extent of change in species composition between sites, and is an important component, especially relevant to explain the factors determining species diversity [2]. Thus, knowledge about the patterns and processes of beta diversity may help us to design better environmental management plans to conserve biological diversity [3].

There are several measures available to assess variation in species composition between sites, and there are some recent proposals of new methodological approaches that contribute to reach a better understanding of beta diversity e.g., [4]. In this work we integrate two of these recent methods: the partitioning of beta diversity into the components of turnover and richness differences [5], and the measurement of dissimilarity in the taxonomic structure of communities [6].

On one hand, partitioning of beta diversity solves the problem related to identical values in traditional dissimilarity indices, for cases where different patterns of species composition occur as a result of two processes: species turnover and nestedness due to richness differences [5, 7–11]. Identifying which one of these processes is the responsible of beta diversity in a region may be crucial to explain biodiversity patterns and to plan conservation strategies [5, 8, 10, 12]. For example, the partitioning of beta diversity has been used recently to identify management strategies for regional biodiversity in boreal lake communities [13], and to assess patterns of temporal beta diversity in agricultural landscapes as a function of long-term land use changes [14].

**Fig 1:**
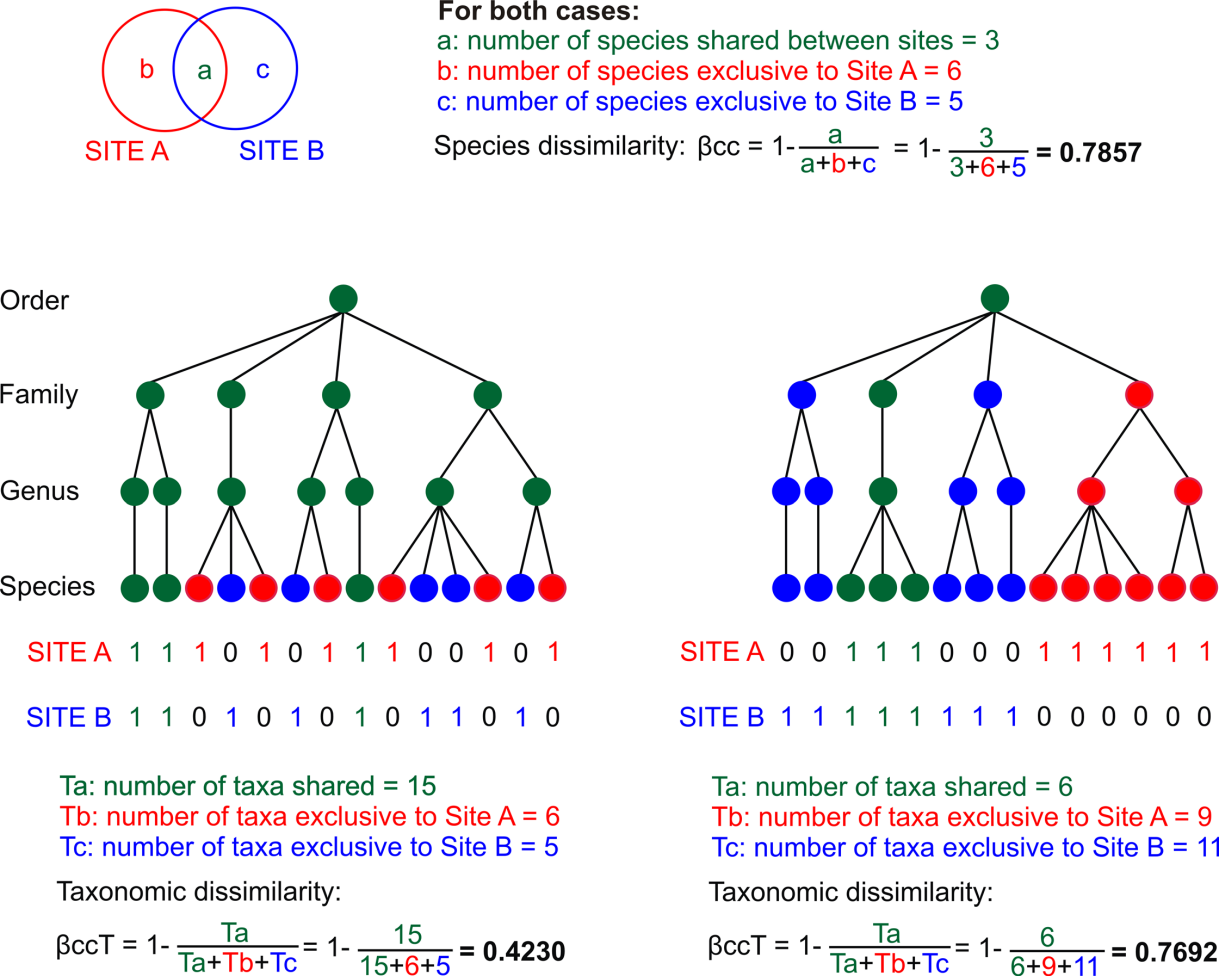
Hypothetical examples showing species and taxonomic dissimilarity. In both cases, the pair of sites has the same species dissimilarity (βcc), but different taxonomic dissimilarity (βccT). For each site species presence (1) or absence (0) is shown. In the taxonomic trees, red circles represent the taxa that are present only in site A, blue circles are taxa found exclusively on site B, and green circles are the taxa shared between sites. Taxonomic dissimilarity is low (βccT = 0.423) when all the genera and families are shared, while it increases (βccT = 0.769) when only one genera and one family are present in both sites.

On the other hand, traditional dissimilarity indices are based on species lists, with or without considering data on their relative abundance, assuming that all species in a community are equally distinct [4]. Taking this limitation into account, there are recent proposals that may widen the scope of dissimilarity indices by including functional or morphological differentiation, genetic distances, taxonomic relatedness or phylogenetic information, to infer about ecological and/or evolutionary processes driving beta diversity e.g., [15]. In particular, dissimilarity in the taxonomic structure (called taxonomic dissimilarity herein) explicitly includes the hierarchical taxonomic classification above species level to measure taxonomic relatedness, as a surrogate of phylogenetic information [6, 16–17]. Basically, taxonomic dissimilarity considers the arrangement of taxa in the Linnaean taxonomic hierarchy as a crude approximation to their evolutionary distinctness [6], and is based on the framework developed by Warwick and Clarke [18]. Taxonomic dissimilarity has advantages over conventional similarity indices, which utilize only data at the species level, since it makes comparisons between sites or areas taking into account higher taxonomic levels (genera, families, orders, etc.). In a hypothetical illustration with pairs of sites that have exactly the same species dissimilarity, two sites may have lower taxonomic dissimilarity than another pair, if the former have more shared higher taxa such as genera and families (Fig 1). Taxonomic dissimilarity also has advantages over phylogenetic beta diversity because it only requires the taxonomical arrangement of species, instead of fully solved phylogenies [6, 18]. In this sense, taxonomic dissimilarity is an easily available indicator of evolutionary dissimilarity between communities, called phylogenetic beta diversity or phylobetadiversity [15, 17, 19–20], especially useful for cases where phylogenies are not available. Both taxonomic and phylogenetic dissimilarity between ecological communities have great potential in research about links between ecology, evolutionary biology, and biodiversity conservation [21–23].

In this work we present a novel approach to study beta diversity patterns that divides the dissimilarity of taxonomic structure between communities, into the components of turnover and richness differences. To accomplish this, we incorporate species and higher taxa composition following the proposal of Bacaro et al. [16]. Thus, the components of dissimilarity are 1) taxon turnover, and 2) dissimilarity due to differences in taxa richness.

Our study is focused on the beta diversity of terrestrial vertebrates (amphibians, reptiles, birds and mammals) in a highly heterogeneous area: the state of Hidalgo, in central Mexico. This area has a complex geological and biogeographical history, as well as an enormous topographic and climatic variation. Therefore, this region harbors high biological diversity and we expect to find notable beta diversity. To assess it, our analyses are based on dissimilarity between ecoregions, as they are spatial study units with potential to assess conservation priorities [24–26].

Our objective is to compare species and taxonomic dissimilarity patterns for terrestrial vertebrates in the ecoregions of Hidalgo, separating the components of turnover and richness differences. In particular, we answer the following questions: 1) Do the dissimilarity due to turnover, and the dissimilarity due to richness differences have the same contribution to total species and taxonomic dissimilarity?, 2) Which ecoregions have the highest dissimilarity?, 3) Do the same dissimilarity patterns between ecoregions occur for the four groups of vertebrates?, and 4) Is there a correlation between species dissimilarity and taxonomic dissimilarity, taking into account their components?

## Materials and Method

### Study area

**Fig 2:**
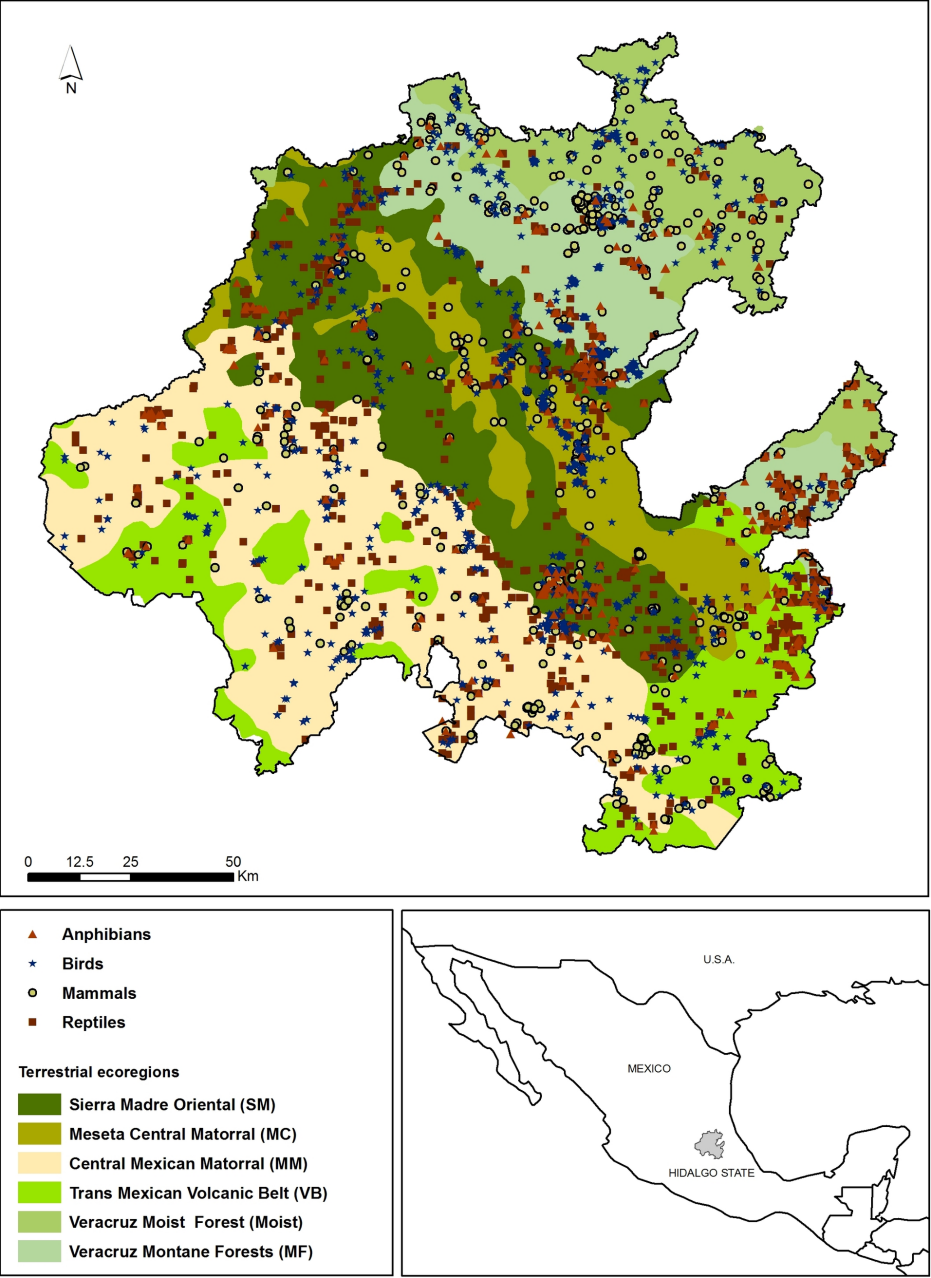
Study area. Spatial distribution of the species records used in this work for terrestrial vertebrates in the ecoregions located in the state of Hidalgo, central Mexico.

We selected the state of Hidalgo, in central Mexico (Fig 2) as study area because of its high biological diversity, which results from the geologic, climatic and biogeographical heterogeneity. The state covers 20,905 km^2^ and an elevation gradient that ranges from 46 to 3,358 m a.s.l. [27]. For this area Morrone [28] proposed four biogeographical provinces: the Mexican Plateau Province (also known as the Altiplano), which belongs to the Nearctic region, the Gulf of Mexico Province that belongs to the Neotropical region, and two provinces of the Halffter’s Mexican Transition Zone *sensu* [29]: the Sierra Madre Oriental, and the Trans-Mexican Volcanic Belt.

We based the analysis of beta diversity in the biotic composition of the ecoregions that occur in the state of Hidalgo. Ecoregions are areas that comprise distinct assemblages of species, with limits that approach the original extent of natural communities before main land use changes [24]. We used the ecoregions delimited by Olson & Dinerstein [25], which correspond to those promoted by the WWF [30] and are used worldwide to evaluate species diversity, endemicity and vulnerability, as well as to identify priority areas for conservation [25, 26].

The ecoregions located in Hidalgo State are (Fig 2): Veracruz Moist Forests (shortened in this work as Moist Forests), Veracruz Montane Forests (Montane Forests), Trans-Mexican Volcanic Belt Pine-Oak Forests (Volcanic Belt), Meseta Central Matorral (Meseta Central), Central Mexican Matorral (Mexican Matorral) and Sierra Madre Oriental Pine-Oak Forests (Sierra Madre).

### Databases

We used the databases of terrestrial vertebrates (amphibians, reptiles, birds, and mammals) from the project Fomix Conacyt-Hidalgo “Biological diversity of the state of Hidalgo” first and second stages (http://citnova.gob.mx/diversidad-biologica-del-estado-de-hidalgo-segunda-fase/; S1 File). We updated those databases for taxonomy and nomenclature up to 2014. The included information results from extensive field work from 2006 to 2010 carried out by specialists in each biological group from the UAEH, and can also be found in other publications [31, 32, 33]. We geo-referenced all records and overlapped them with the ecoregions using the ArcGis 10.1 program [34] to get species lists per ecoregion. We used these lists for all analyses in this work.

We used different sources to determine the taxonomic structure of each vertebrate group and the taxonomic levels used for calculating taxonomic beta diversity. For amphibians and reptiles, we used the catalogs of taxonomical authorities of CONABIO [35, 36]; for mammals we followed the classification of Ramírez-Pulido et al. [37]; and for birds we used the information available in the AOU [38] web page and the phylogenetic arrangement of Stefan Hintsche [39].

### Ethics Statement

Databases include records of field samplings done on public and private lands, with the corresponding permission of owners. Field sampling was authorized by the Secretaría del Medio Ambiente y Recursos Naturales (SEMARNAT, Mexican Council for the Environment and Natural Resources), which legislates scientific field samplings in Mexico, through permissions FAUT-0052 and SGPA/DGVS/02726/10 for amphibians and reptiles, FAUT-0221 and FAUT-0232 for mammals. Birds were not collected. We did not perform any other activities that required specific permissions. For field sampling in Mexico, the approval by an Institutional Animal Care and Use Committee (IACUC) or equivalent animal ethics committee is not required.

### Data analysis

To assess inventories’ completeness for each group of vertebrates at each ecoregion, we calculated the sample coverage [40], considering the total number of records per species, and the number of species with one and two records.

We partitioned beta diversity following the procedure of Carvalho et al. [8, 9], which is based on the approach of Baselga [5]. According to this method, total dissimilarity (βcc) is 1 minus the similarity coefficient of Jaccard. This total dissimilarity is divided into two components: the dissimilarity due to turnover (β.3) and the dissimilarity due to richness differences (βrich). We did this partitioning both for dissimilarity in species composition, and for dissimilarity in taxonomic structure considering the composition of higher taxa. For this last case, we used the method of Bacaro et al. [16], so that total taxonomic dissimilarity, herein βccT (1-Δ_T_ *sensu* Bacaro et al. [16]) equals the dissimilarity of Jaccard coefficient but taking into account higher taxa. Taxonomic dissimilarity is measured as: βccT = 1-(Ta/Ta+Tb+Tc), where Ta is the total number of taxa shared between two communities, Tb is the number of taxa present only in the first community but absent in the second, and Tc is the number of taxa present exclusively in the second community (Fig 1). Values of βccT range from zero when the taxonomic structure of both communities is identical, to 1 when the taxonomic structure is totally different [6]. Taxonomic dissimilarity measures the proportion represented by not-shared taxa from the total number of taxa in the two communities. Therefore, partitioning of βccT with the procedure of Carvalho et al. [8] gives one component of dissimilarity due to taxon turnover (β.3T) and one component of dissimilarity due to difference in taxa richness (βrichT).

To calculate total taxonomic dissimilarity and its components we used as many taxonomic levels as possible. For amphibians: order, family, subfamily, genus and species; and for reptiles: subclass, order, suborder, infraorder, superfamily, family, subfamily, genus and species [35, 36]. For birds: subclass, infraclass, parvoclass, superdivision, division, subdivision, infradivision, superorder, order, suborder, superfamily, family, subfamily, genus and species [38, 39]. Finally, for mammals: order, suborder, infraorder, superfamily, family, subfamily, tribe, genus and species [37].

We did all the analysis of dissimilarity partitioning in the R program [41], using the script of Carvalho et al. [9]. First, to assess if the two components of total species and taxonomic dissimilarity have equal contributions, we used multiple-site measures, *sensu* Diserud & Ødegaard [42], including all the ecoregions that occur in Hidalgo. Second, to detect which ecoregions have the highest values of species and taxonomic dissimilarity for each biological group, we used pairwise comparisons indices between ecoregions, as described above (βcc, βrich, β.3, βccT, βrichT, and β.3T). Then, to spatially represent the values of these indices we used multivariate ordinations of non-metric multidimensional scaling (NMDS). Finally, we did Pearson correlations between species and taxonomic dissimilarity, as well as their components, for each biological group. The NMDS and correlations were done in the program Past 3.07 [43].

## Results

Amphibians are the least represented group in our databases, with 1,564 records belonging to 50 species, while birds had the highest number of records: 31,827 from 515 species (Table 1). The least complete inventories stand for reptiles and birds in the Moist Forests (76.72 and 90.64%, respectively). The rest of the inventories are at least 94% complete according to their sample coverage, based on species records per ecoregion (Table 1). Overall, these results indicate that a high proportion of the species that occur in the different ecoregions were recorded, and therefore it is possible to do reliable analyses of beta diversity.

**Table 1:** Number of species (S) and records (R) of terrestrial vertebrates at each ecoregion in Hidalgo, Mexico, and the percentage of inventory completeness (Com) calculated as the sample coverage [39].

	Amphibians			Reptiles			Birds			Mammals		
	S	R	Com	S	R	Com	S	R	Com	S	R	Com
Montane Forests	29	405	98.50	66	613	94.30	267	4,125	98.25	69	1,270	98.74
Moist forests	21	131	94.70	53	146	76.72	217	939	90.64	32	237	97.49
Volcanic Belt	18	192	96.90	30	465	96.56	189	1,026	94.06	22	156	95.53
Mexican Matorral	13	166	99.40	47	1,012	97.82	211	4,009	98.83	48	554	97.84
Meseta Central	9	106	99.10	36	188	95.24	213	13,240	99.63	45	973	98.76
Sierra Madre	28	564	98.76	58	1,317	99.16	309	8,488	99.26	47	366	96.46
Total	50	1,564		131	3,741		515	31,827		104	3,556	

**Fig 3:**
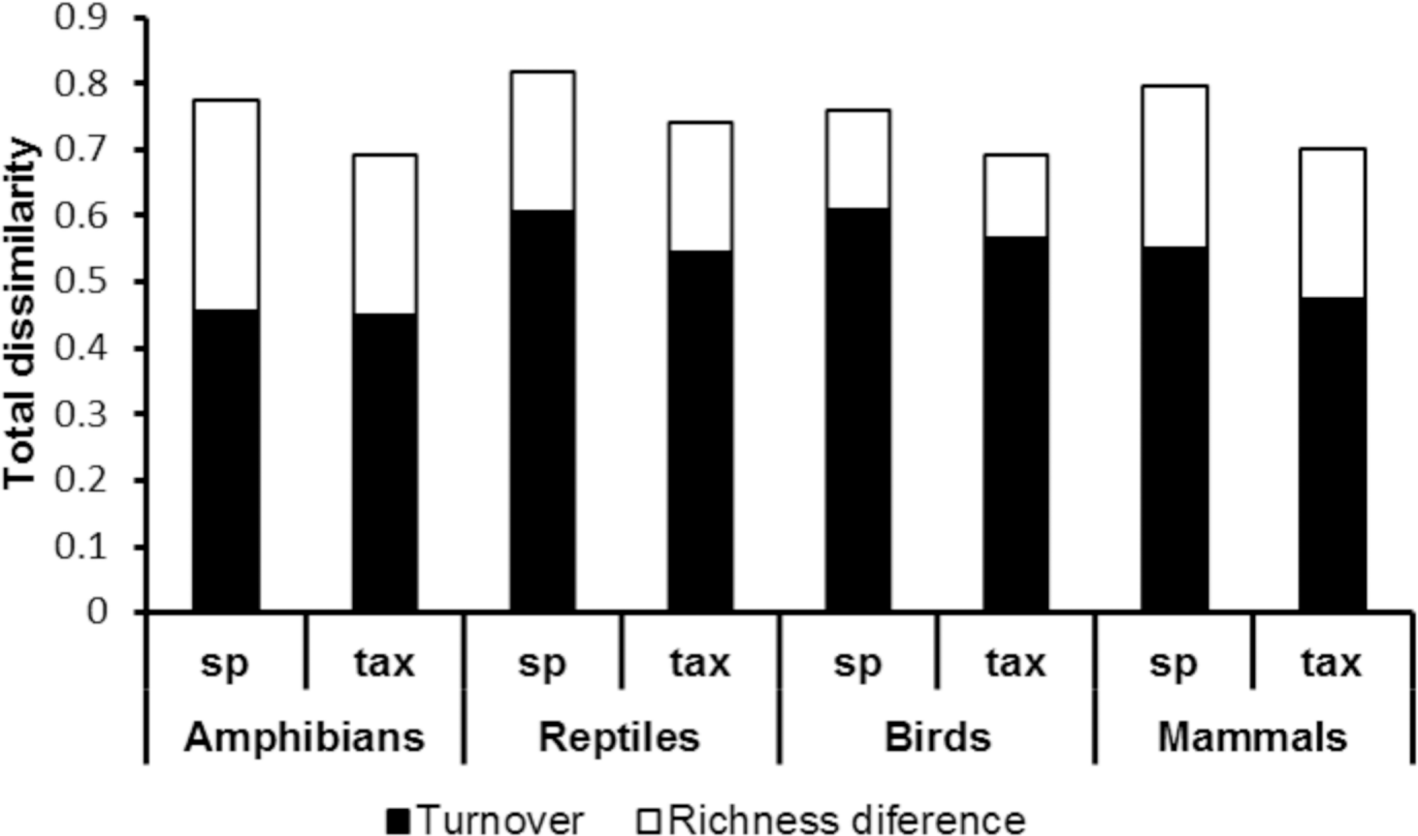
Multiple-site dissimilarity of terrestrial vertebrates. Species and taxonomic total dissimilarity, and their turnover and richness difference components among six ecoregions in Hidalgo, Mexico.

The four groups of vertebrates had high values of total dissimilarity among ecoregions, and the dissimilarity based on their taxonomic structure was always lower than species dissimilarity (Fig 3). Birds had the lowest dissimilarity (75.87 and 69.04% for species and taxonomic dissimilarity, respectively), while the highest values were recorded for reptiles (81.79 and 74.18% for species and taxonomic dissimilarity, respectively, Fig 3).

For the first question, regarding the contribution of dissimilarity components, when we partitioned total dissimilarity in the four vertebrate groups, we found that turnover consistently had a higher contribution than the richness difference component. This pattern occurred both for species and taxonomic dissimilarity. Turnover accounted for 58.76 to 80.22% of species dissimilarity, and for 64.99 to 82.14% of taxonomic dissimilarity. In both cases, the lowest values corresponded to amphibians, and the highest to birds (Fig 3). Thus, the maximum contribution of richness differences was 41% for amphibians.

**Fig 4:**
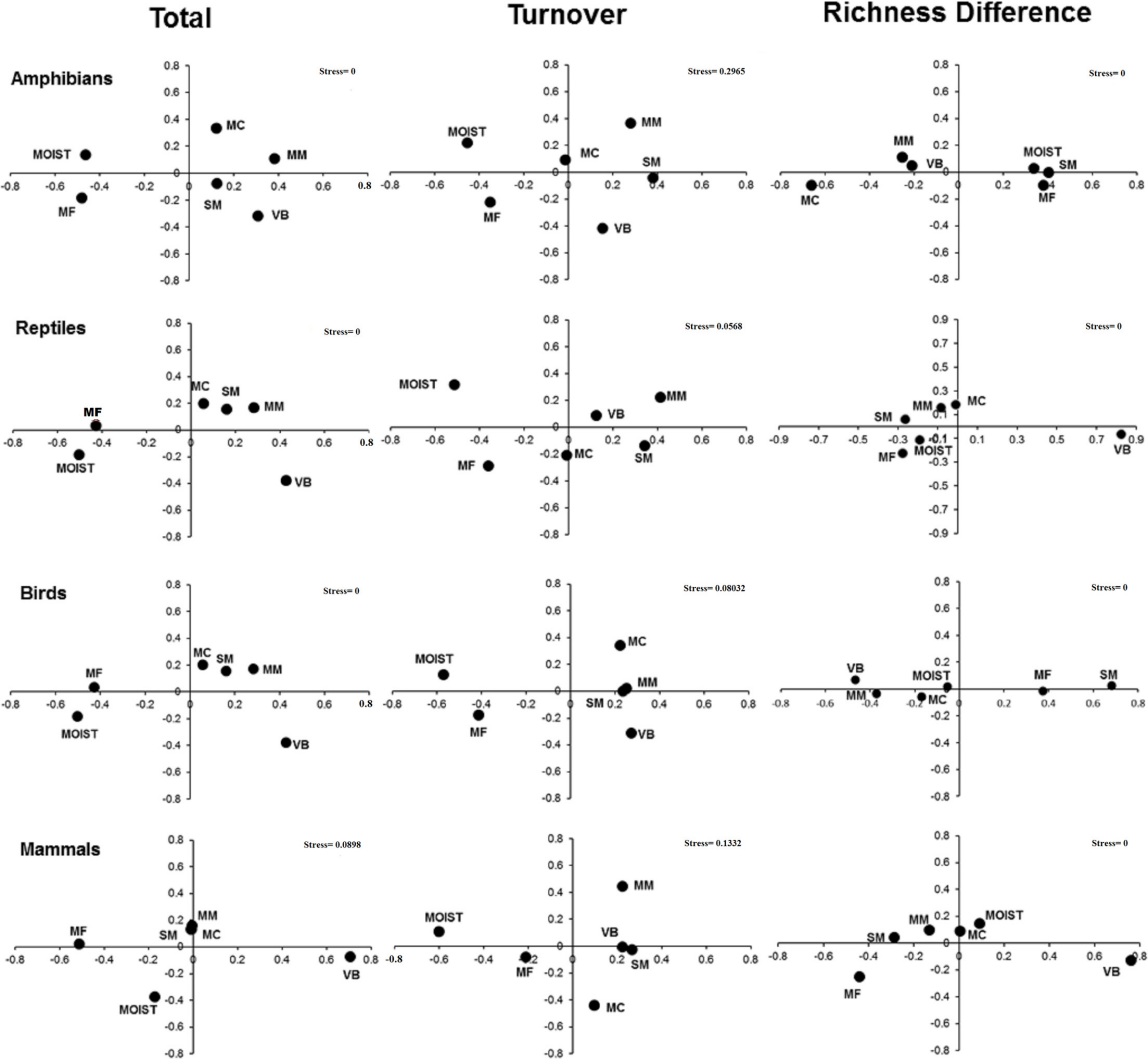
NMDS results for the four terrestrial vertebrate groups. Dissimilarity in the taxonomic composition (βccT, β.3T and βrichT) determines the relative position of the six ecoregions of Hidalgo State, Mexico. Moist: Moist Forests, MF: Montane Forests, VB: Volcanic Belt, MC: Meseta Central, MM: Mexican Matorral, SM: Sierra Madre.

With respect to the second question, as with multiple comparisons, in pairwise comparisons between ecoregions species dissimilarity was higher than taxonomic dissimilarity. The highest values of species and taxonomic dissimilarity correspond to reptiles and birds between the Moist Forest and the Volcanic Belt ecoregions (S1 Table). The differences in composition among ecoregions are clearly shown in the NMDS graphs (Fig 4). The NMDS showed similar results for species and taxonomic dissimilarity, thus we only provide here the results of taxonomic dissimilarity (Fig 4).

Regarding the third question about differences among vertebrates, we found that turnover had a consistently higher contribution than richness differences for the four vertebrate groups, in species and taxonomic dissimilarity (S1 Table). The results suggest analogous total taxonomic dissimilarity and turnover for amphibians, reptiles and birds, given that the spatial distributions of ecoregions in the graphs are similar: Moist Forest and Montane Forest are clearly different in composition from the other four ecoregions (Fig 4). However, the dissimilarity due to richness differences varies for these vertebrates. In contrast, mammals had different trends in total, turnover and richness difference dissimilarities. For total dissimilarity the scrublands (Meseta Central and Mexican Matorral) and the Sierra Madre had similar taxonomic composition, while the Volcanic Belt, Montane Forest, and Moist Forest ecoregions differ. However, taxonomic turnover was low between Volcanic Belt and the Sierra Madre ecoregions, but the Mexican Matorral and the Meseta Central have high turnover (Fig 4).

**Fig 5:**
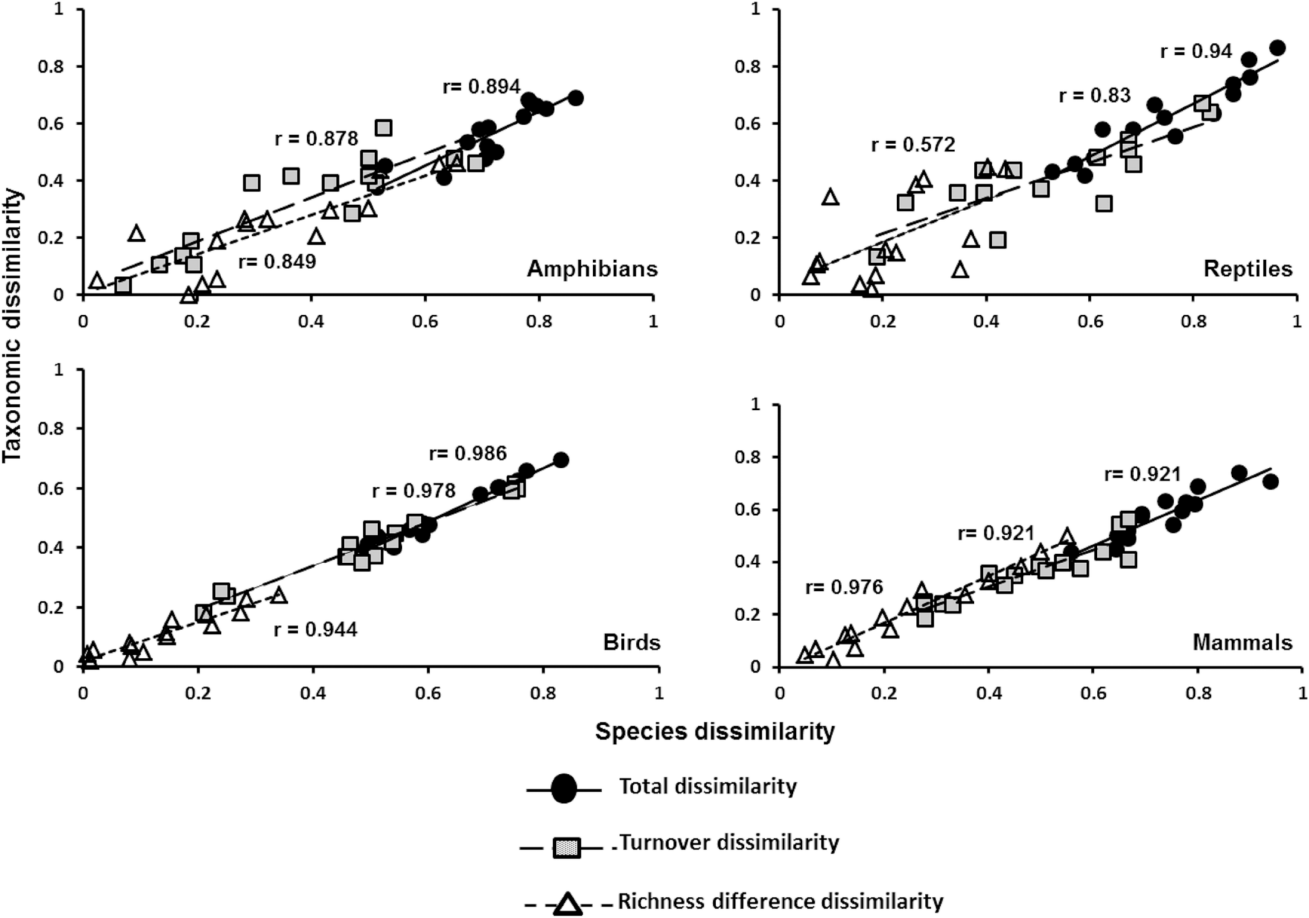
Relationships between species and taxonomic dissimilarity. Pearson correlations for total dissimilarity and its components of turnover and richness differences in pairwise comparisons of ecoregions, for each vertebrate group.

For the fourth question related to the relationship between dissimilarity types, we found that species dissimilarity and the dissimilarity based on the taxonomic structure are positively correlated (P<0.05) for the four vertebrate groups (Fig 5). The highest correlation coefficients correspond to total dissimilarity and turnover dissimilarity in birds, while the lowest values were found in total dissimilarity and its components for amphibians, as well as the two components for reptiles (Fig 5).

## Discussion

Our results show that species dissimilarity is higher than taxonomic dissimilarity for terrestrial vertebrates in the ecoregions of our study area, because when incorporating higher taxa into the analysis more shared elements are included, and communities tend to homogenize their composition.

### Contribution of dissimilarity components

Not surprisingly, turnover has a more important contribution to total dissimilarity than richness differences, and birds had the highest turnover value while amphibians had the lowest one. This prominence of turnover coincides with beta diversity patterns of vertebrates in the Isthmus of Tehuantepec [44], amphibians in Brazilian Mata Atlantica [45], reproductive birds and lizards in flooded islands of East China [11], and with temporal beta diversity of birds in agricultural fields in France [14]. The spatial scale may have an influence in all these results because at small scales environmental filters exclude species that lack physiological tolerance to climatic or environmental local conditions [46]. In contrast, when the study area is broader, biogeographic processes influence species turnover and nestedness due to richness differences [23]. This occurs for example in global patterns of amphibian distributions, where the relative importance of beta diversity components changes with latitude: turnover is more important below parallel 37, while above this richness differences become more important [47]. Also, in America nestedness due to richness differences for amphibians increases as we approach to the poles, but it decreases for birds and mammals [48]. However, in the Mexican Transition Zone, biogeographic processes may influence turnover even in small areas.

Differences in species and taxa composition between ecoregions for reptiles, birds and mammals may be more influenced by environmental heterogeneity (differences in ecological conditions and vegetation types that lead to turnover) and historical factors than by differences in the number of species. But for amphibians, richness differences are more important in determining beta diversity than turnover between ecoregions. Other studies with vertebrates have explored possible causes. For example, in a global study with amphibians Baselga et al. [47] found that turnover was related to variation in elevation and temperature, while richness differences were related to evapotranspiration, annual precipitation and mean temperature. Also, Melo et al. [46] found that bird and mammal beta diversity in America is related to environmental heterogeneity, measured as elevation range, and this variable is responsible for up to 52% of bird beta diversity and 21% of mammal beta diversity. Moreover, for mammals in Europe beta diversity is related to topographic heterogeneity, mean annual temperature, as well as real and potential evapotranspiration [49]. Similarly, for terrestrial mammals in Mexico the highest beta diversity occurs in the Transvolcanic Belt, which is the most heterogeneous region of the country, while the lowest beta diversity corresponds to the most homogeneous region, i.e. the Yucatan Peninsula [50]. This pattern was later confirmed for terrestrial vertebrates [51], supporting the idea that environmental heterogeneity is a key factor determining beta diversity [46, 49]. Thus, although we do not have data on environmental heterogeneity at the scale of ecoregions, we assume that this factor may be crucial in our study area given the high proportion of turnover found in our results, and how turnover is related with heterogeneity as described in these studies.

### Dissimilarity between ecoregions

The ecoregions in Hidalgo have different origins and geological histories, which result in the current complex topography, climatic heterogeneity and variation in vegetation types [27–29]. For example, the Meseta Central ecoregion is isolated form other arid zones, and in Hidalgo it is surrounded by the Sierra Madre Oriental. This Sierra Madre ecoregion harbors conifer forests, mainly pine-oak forests, and goes from North U.S.A. to Southern Mexico, and connects with the Volcanic Belt [30]. The Volcanic Belt is located in central Mexico and has several active and inactive volcanoes, including 13 of the highest mountains in the country. This intense volcanic and orogenic activity has allowed the formation of microhabitats, which promoted taxa radiation and speciation [28–29]. The Mexican Matorral is one of the most extensive ecoregions in the country, and the largest in Hidalgo. It emerged from superficial folds of Cretaceous sediments, resulting in a region with abrupt topography that includes valleys, canyons and ravines [28]. In Hidalgo this ecoregion includes three types of scrubland. Finally, the Moist Forest and Montane Forest ecoregions are located in the northeastern portion of the state, and include tropical perennial and sub-perennial forests, as well as the northern mountain cloud forests, that encompass high plant and animal richness and are currently threatened by human activities [30].

In our results, these two ecoregions (Moist Forest and Montane Forest) resulted with similar taxonomic composition because of the Neotropical affinity of their fauna, while they are located on the other side than Meseta Central, Sierra Madre Oriental, Volcanic Belt and Mexican Matorral in the NMDS graphs (Fig 3). These four ecorregions are more related with North America and thus their fauna has Nearctic affinity [28–29]. Thus, dissimilarity is clearly influenced by the biogeographic origin of the biota.

### Differences among vertebrates

Contrary to previous studies [44, 46, 48, 51–53], we found that reptiles and mammals have the highest values of dissimilarity, while amphibians and birds have the lowest, although differences are small. For example, in the Isthmus of Tehuantepec in southeastern Mexico, where there are no altitudinal differences birds had the highest values of beta diversity, while amphibians had the lowest values [44]. Also, at the scale of the American continent birds had higher values of beta diversity than mammals [46]. Other studies show that beta diversity may be higher for amphibians and reptiles than for mammals and birds [48, 51–53]. In most cases, these results seem to be related with the dispersal abilities and niche limitations, because exothermal organisms (amphibians and reptiles) had in general lower dispersal capacities and stronger niche limitations than birds and mammals [48, 52–53].

### Relationship between dissimilarity types

Correlations between species and taxonomic dissimilarities indicate that the number of species that may be considered in conservation efforts is related to the number of higher taxa. This is important because it suggests that, in our study area, if we focus on the species level for conservation, we are not only protecting current biological diversity, but also the taxonomic and evolutionary diversity of each ecoregion will be preserved, which is the result of millions of years of evolution.

Izsac & Price [6] also found this relationship between beta diversity and taxonomic beta diversity for different echinoderm communities in Asia. Furthermore, we think that it would be possible to find positive relationships between taxonomic beta diversity, phylogenetic and functional beta diversities for terrestrial vertebrates, in a similar way that has been found for freshwater fish in watersheds [54], and for birds in environmental gradients in France [55]. However, sometimes, taxonomic beta diversity is not related in any way to other measures of beta diversity, as occurs for estuarine tropical fish in Mexico, where functional beta diversity is low even when taxonomic beta diversity is high, because of the dominance of few functionally similar species and the low proportion of specialists [56].

### Concluding remarks

This extraordinarily vast heterogeneity and environmental complexity requires complementary approaches so current biodiversity can be understood form different perspectives. In this paper we have shown trends in species and taxonomic beta diversity and their components. However, including other aspects such as phylogenetic and functional diversity measures will give a broader overview of biodiversity. Also, incorporating information of different biological groups and spatial scales would be desirable. Our results for terrestrial vertebrates may be helpful to assess risks and implications of beta diversity loss and the consequent homogenization of biotas, especially for ecological integrity and functioning at each ecoregion. For example, Brazilian Atlantic mixed forests have higher phylobetadiversity than other forest types because of the presence of temperate and tropical taxa that make them unique, and therefore this region requires the implementation of different conservation strategies [19]. This result from the Brazilian mixed forest coincides with our results of vertebrate beta diversity in the Mexican Transition Zone, especially when we include the taxonomic structure and highlight the importance of species and higher taxa turnover among ecoregions. As a conservation strategy of vertebrates, we believe that its necessary to review in future research projects the design of the current system of natural protected areas in Hidalgo, in order to guarantee the inclusion of relevant representative areas at the ecoregion level. In particular, our results indicate that to conserve amphibians the richest sites within each ecoregion require protection, because of the high contribution of dissimilarity due to richness differences between ecoregions. But for the conservation of reptiles, birds and mammals a system of protected areas, that include most of the heterogeneity within and among ecoregions, is needed. These local protection actions will warrant regional biological diversity.

## Supporting Information

S1 File Data used in this paper. Number of records of each species considered in this study, for the four groups of terrestrial vertebrates in the six ecoregions of Hidalgo, Mexico.
(XLSX)

S1 Table Dissimilarity between ecoregions. Pairwise dissimilarity values between ecoregions in Hidalgo, Mexico, for the species and taxonomic composition of terrestrial vertebrates.
(DOCX)
