# Supplementary material for: Beta Diversity in a Highly Heterogeneous Area: Disentangling Species and Taxonomic Dissimilarity for Terrestrial Vertebrates
Source: PLoS One. 2016 Aug 8;11(8):e0160438. doi: 10.1371/journal.pone.0160438 (PMC4976865; doi:10.1371/journal.pone.0160438)
Supplement: S1 Table — Pairwise dissimilarity values between ecoregions in Hidalgo, Mexico, for the species and taxonomic composition of terrestrial vertebrates. (DOCX) [file pone.0160438.s002.docx]

***S1* *SUPPORTING INFORMATION***

**Beta Diversity in a Highly Heterogeneous Area: Disentangling Species and Taxonomic Dissimilarity for Terrestrial Vertebrates**

Jaime M. Calderón-Patrón, Irene Goyenechea, Raúl Ortiz-Pulido, Jesús Castillo-Cerón, Norma Manriquez, Aurelio Ramírez-Bautista, Alberto Rojas-Martínez, Gerardo Sánchez-Rojas, Iriana Zuria, Claudia E. Moreno.

**S1 Table. Pairwise dissimilarity values between ecoregions in the state of Hidalgo, Mexico, for the species and taxonomic composition of terrestrial vertebrates.** Total dissimilarity and its components (turnover and richness difference) are included. The highest values are shown in red and the lowest in blue. See Methods for more explanation.

|  | Species dissimilarity | | | Taxonomic dissimilarity | | |
| --- | --- | --- | --- | --- | --- | --- |
|  | Total dissimilarity (βcc) | Turnover dissimilarity (β.3) | Richness difference dissimilarity (βrich) | Total dissimilarity (βccT) | Turnover dissimilarity (β.3T) | Richness difference dissimilarity (βrichT) |
| **Amphibians** |  |  |  |  |  |  |
| MontaneForest/MoistForest | 0.5294 | 0.2941 | 0.2353 | 0.4507 | 0.3944 | 0.0563 |
| MontaneForest/VolcanicBelt | 0.7949 | 0.5128 | 0.2821 | 0.6620 | 0.3944 | 0.2676 |
| MontaneForest/MexicanMatorral | **0.8649** | 0.4324 | 0.4324 | **0.6901** | 0.3944 | 0.2958 |
| MontaneForest/Meseta Central | 0.8125 | 0.1875 | 0.6250 | 0.6508 | 0.1905 | 0.4603 |
| MontaneForest/SierraMadre | 0.6744 | 0.6512 | **0.0233** | 0.5333 | 0.4800 | 0.0533 |
| MoistForest/VolcanicBelt | 0.7813 | **0.6875** | 0.0938 | 0.6812 | 0.4638 | 0.2174 |
| Moist Forest/Mexican Matorral | 0.7857 | 0.5000 | 0.2857 | 0.6716 | 0.4179 | 0.2537 |
| MoistForest/MesetaCentral | 0.6957 | 0.1739 | 0.5217 | 0.5789 | 0.1404 | 0.4386 |
| Moist Forest/Sierra Madre | 0.7105 | 0.5263 | 0.1842 | 0.5867 | **0.5867** | **0.0000** |
| Volcanic Belt/Mexican Matorral | 0.7083 | 0.5000 | 0.2083 | 0.5200 | 0.4800 | 0.0400 |
| VolcanicBelt/MesetaCentral | 0.7727 | 0.3636 | 0.4091 | 0.6250 | 0.4167 | 0.2083 |
| VolcanicBelt/SierraMadre | **0.5161** | 0.1935 | 0.3226 | **0.3750** | 0.1071 | 0.2679 |
| MexicanMatorral/MesetaCentral | 0.7059 | 0.4706 | 0.2353 | 0.4762 | 0.2857 | 0.1905 |
| Mexican Matorral/Sierra Madre | 0.6333 | 0.1333 | 0.5000 | 0.4107 | 0.1071 | 0.3036 |
| Meseta Central/Sierra Madre | 0.7241 | **0.0690** | **0.6552** | 0.5000 | **0.0370** | **0.4630** |
|  |  |  |  |  |  |  |
| **Reptiles** |  |  |  |  |  |  |
| MontaneForest/Moist Forest | 0.6071 | 0.4524 | 0.1548 | 0.4797 | 0.4390 | 0.0407 |
| Montane Forest/Volcanic Belt | 0.9080 | 0.5057 | 0.4023 | 0.8220 | 0.3729 | **0.4492** |
| MontaneForest/MexicanMatorral | 0.8776 | 0.6735 | 0.2041 | 0.7045 | 0.5455 | 0.1591 |
| MontaneForest/MesetaCentral | 0.7654 | 0.3951 | 0.3704 | 0.5556 | 0.3590 | 0.1966 |
| MontaneForest/SierraMadre | 0.7449 | 0.6735 | 0.0714 | 0.6202 | 0.5116 | 0.1085 |
| MoistForest/VolcanicBelt | **0.9620** | 0.6835 | 0.2785 | **0.8644** | 0.4576 | 0.4068 |
| MoistForestMexicanMatorral | 0.9101 | **0.8315** | 0.0787 | 0.7612 | 0.6418 | 0.1194 |
| Moist Forest/MesetaCentral | 0.8400 | 0.6133 | 0.2267 | 0.6333 | 0.4833 | 0.1500 |
| MoistForest/SierraMadre | 0.8776 | 0.8163 | **0.0612** | 0.7372 | **0.6715** | 0.0657 |
| VolcanicBelt/MexicanMatorral | 0.6842 | 0.4211 | 0.2632 | 0.5783 | 0.1928 | 0.3855 |
| VolcanicBelt/MesetaCentral | 0.7255 | 0.6275 | 0.0980 | 0.6667 | 0.3218 | 0.3448 |
| VolcanicBelt/SierraMadre | 0.6250 | **0.1875** | **0.4375** | 0.5795 | **0.1364** | 0.4432 |
| MexicanMatorral/MesetaCentral | 0.5714 | 0.3929 | 0.1786 | 0.4583 | 0.4375 | **0.0208** |
| MexicanMatorral/SierraMadre | **0.5286** | 0.3429 | 0.1857 | 0.4300 | 0.3600 | 0.0700 |
| MesetaCentral/Sierra Madre | 0.5909 | 0.2424 | 0.3485 | **0.4184** | 0.3265 | 0.0918 |
|  |  |  |  |  |  |  |
| **Birds** |  |  |  |  |  |  |
| MontaneForest/MoistForest | 0.6012 | 0.4566 | 0.1445 | 0.4754 | 0.3713 | 0.1041 |
| MontaneForest/VolcanicBelt | 0.7541 | 0.5410 | 0.2131 | 0.6265 | 0.4498 | 0.1767 |
| MontaneForest/MexicanMatorral | 0.6904 | 0.5370 | 0.1534 | 0.5784 | 0.4202 | 0.1583 |
| MontaneForest/MesetaCentral | 0.7200 | 0.5760 | 0.1440 | 0.6039 | 0.4863 | 0.1176 |
| MontaneForest/SierraMadre | 0.5672 | 0.4627 | 0.1045 | 0.4614 | 0.4113 | 0.0501 |
| MoistForest/VolcanicBelt | **0.8300** | 0.7493 | 0.0807 | **0.6959** | **0.6164** | 0.0795 |
| MoistForest/MexicanMatorral | 0.7701 | **0.7529** | 0.0172 | 0.6574 | 0.5992 | 0.0583 |
| MoistForest/MesetaCentral | 0.7536 | 0.7420 | 0.0116 | 0.6137 | 0.5914 | **0.0223** |
| MoistForest/SierraMadre | 0.7233 | 0.5000 | 0.2233 | 0.6032 | 0.4627 | 0.1405 |
| VolcanicBelt/MexicanMatorral | 0.5401 | 0.4599 | 0.0803 | 0.4007 | 0.3726 | 0.0281 |
| VolcanicBelt/MesetaCentral | 0.5895 | 0.5053 | 0.0842 | 0.4452 | 0.3754 | 0.0698 |
| VolcanicBelt/SierraMadre | 0.5892 | 0.2493 | **0.3399** | 0.4829 | 0.2393 | **0.2436** |
| MexicanMatorral/Meseta Central | **0.4911** | 0.4840 | **0.0071** | **0.3946** | 0.3508 | 0.0438 |
| MexicanMatorral/SierraMadre | 0.4928 | **0.2087** | 0.2841 | 0.4103 | **0.1824** | 0.2279 |
| MesetaCentral/SierraMadre | 0.5128 | 0.2393 | 0.2735 | 0.4364 | 0.2542 | 0.1822 |
|  |  |  |  |  |  |  |
| **Mammals** |  |  |  |  |  |  |
| MontaneForest/MoistForest | 0.6667 | 0.3111 | 0.3556 | 0.5191 | 0.2443 | 0.2748 |
| MontaneForest/VolcanicBelt | 0.8791 | 0.3297 | **0.5495** | **0.7395** | 0.2375 | **0.5019** |
| MontaneForest/MexicanMatorral | 0.6931 | 0.4950 | 0.1980 | 0.5754 | 0.3860 | 0.1895 |
| MontaneForest/MesetaCentral | 0.6939 | 0.4490 | 0.2449 | 0.5842 | 0.3513 | 0.2330 |
| MontaneForest/SierraMadre | 0.6471 | 0.5098 | 0.1373 | 0.5000 | 0.3688 | 0.1312 |
| MoistForest/VolcanicBelt | **0.9394** | **0.6667** | 0.2727 | 0.7085 | 0.4121 | 0.2965 |
| MoistForest/MexicanMatorral | 0.7952 | 0.6506 | 0.1446 | 0.6198 | 0.5455 | 0.0744 |
| MoistForest/MesetaCentral | 0.7692 | **0.6667** | 0.1026 | 0.5957 | **0.5652** | **0.0304** |
| MoistForest/SierraMadre | 0.7529 | 0.5412 | 0.2118 | 0.5436 | 0.3983 | 0.1452 |
| VolcanicBelt/MexicanMatorral | 0.7385 | **0.2769** | 0.4615 | 0.6318 | 0.2488 | 0.3831 |
| VolcanicBelt/MesetaCentral | 0.8000 | 0.4000 | 0.4000 | 0.6866 | 0.3582 | 0.3284 |
| VolcanicBelt/SierraMadre | 0.7778 | 0.2778 | 0.5000 | 0.6291 | **0.1878** | 0.4413 |
| MexicanMatorral/MesetaCentral | 0.6667 | 0.6173 | **0.0494** | 0.4912 | 0.4425 | 0.0487 |
| MexicanMatorral/SierraMadre | 0.6437 | 0.5747 | 0.0690 | 0.4496 | 0.3782 | 0.0714 |
| MesetaCentral/SierraMadre | **0.5570** | 0.4304 | 0.1266 | **0.4367** | 0.3144 | 0.1223 |
